# Pan-genome analysis of wax apple (*Syzygium samarangense*) and its association with fruit size and cold tolerance

**DOI:** 10.3389/fpls.2026.1703197

**Published:** 2026-02-03

**Authors:** Xing Long, Liang Li, Ren Fang, Ji Zhang, Ping Zhou, Zhenyu An, Wenzhong Tang, Jinyan Yao, Xiuqing Wei

**Affiliations:** 1 Horticultural Research Institute, GuangXi Academy of Agricultural Sciences, Nanning, China; 2 Fruit Research Institute, Fujian Academy of Agricultural Sciences, Fuzhou, Fujian, China

**Keywords:** cold-tolerance, fruit size, pangenome, population genome, selection analysis, *Syzygium samarangense*

## Abstract

**Introduction:**

Wax apple (Syzygium samarangense) is a tropical fruit crop of high economic value, in which fruit size and cold tolerance are key traits affecting cultivation range and market quality. However, the genetic basis underlying these traits remains poorly understood at the genome-wide level.

**Methods:**

We constructed the first wax apple pangenome using whole-genome resequencing data from 27 accessions. Novel non-redundant sequences were identified and annotated, and genes were classified based on presence/absence variation (PAV). Population structure was inferred using both PAV and single-nucleotide polymorphism (SNP) data. Structural variants (SVs) were detected genome-wide, and candidate genes associated with fruit size and cold tolerance were identified by integrating PAV, SNP-based XP-CLR, and SV-based F_ST analyses.

**Results:**

The pangenome contained 69 Mb of novel non-redundant sequences and 707 newly predicted genes. PAV analysis classified 35,468 genes as core, 10,789 as dispensable, and 364 as private. Population structure analyses consistently divided the accessions into three subgroups, indicating a multi-lineage domestication history. We identified 44,567 SVs, including 9,999 duplications, 34,593 deletions, and 65 insertions. Integrative selective sweep analyses revealed candidate genes associated with fruit size and cold tolerance. An S-adenosylmethionine-dependent methyltransferase gene was located within a cold-tolerance-related selective sweep region. Genes from the Mol family, as well as genes involved in abscisic acid metabolism and quercetin 3′-O-glucosyltransferase activity, were significantly enriched in large-fruited accessions. Additionally, SVs showed strong genetic differentiation in diterpene phytoalexin biosynthesis genes among cold-tolerant lines.

**Discussion:**

This study provides the first pangenome resource for wax apple and reveals extensive genomic variation associated with key agronomic traits. The identified candidate genes and structural variants offer insights into the genetic mechanisms underlying fruit size and cold tolerance and provide a genomic foundation for trait improvement and molecular breeding of wax apple.

## 1 Introduction

*S. samarangense*, commonly known as wax apple, Java apple, or water apple, is a typical tropical evergreen fruit tree belonging to the genus *Syzygium* of the family Myrtaceae. Native to the Andaman and Nicobar Islands and the Malaysian Archipelago (Shü et al., 2011; Khandaker and Boyce, 2016), it is now widely cultivated in regions such as Taiwan (China), Thailand, Indonesia, and Malaysia (Zen-hong Shü et al., 2008). With its vibrant coloration (typically deep red, pink, or milky white), bell- or pear-shaped appearance, and crisp, juicy texture, wax apple is valued for both its ornamental and edible qualities, occupying a significant niche in the high-end fruit markets of Southeast Asia and southern China. In recent years, increasing consumer demand for functional fruits has drawn attention to wax apple due to its rich content of bioactive compounds, including proteins, dietary fiber, sugars, vitamins, flavonoids, and phenolic acids. These constituents offer potential health benefits such as antioxidant, anti-inflammatory, and metabolic regulatory effects (Banadka et al., 2022), further spurring research interest in their nutritional and medicinal applications.

Despite its commercial potential, the industrial development of wax apple still faces several challenges. On one hand, as a typical tropical species, it is highly sensitive to low-temperature stress, and its poor cold tolerance severely limits its introduction and cultivation in subtropical regions. On the other hand, fruit size, a key trait determining market value, is influenced by a complex genetic regulatory network and environmental interactions, resulting in significant variation in single-fruit weight among different cultivars (ranging from 28 to 100 g, with a maximum up to 200 g) (Huang, 2017). Currently, few studies focus on the genetic basis of cold tolerance or fruit size in wax apples. In addition, wax apple exhibits complex genomic characteristics, with existing germplasm resources including both diploid and tetraploid types, which has delayed progress in genetic analysis and molecular breeding compared to other major fruit crops.

Recent advances in high-throughput sequencing have opened new avenues for wax apple genomics. Reference genomes for both diploid (Wei et al., 2023) and tetraploid (Zhang et al., 2024) varieties have been published. However, a single reference genome cannot capture the full genetic diversity of a species. In contrast, the construction of a pan-genome, which integrates population-scale sequence variations such as PAVs and SVs, offers a more comprehensive genomic resource for investigating the genetic basis of important traits (Vernikos et al., 2015). In this study, we constructed the first pan-genome of wax apple using whole-genome resequencing data from diverse cultivars. We systematically identified inter-individual PAVs and SVs, and, by integrating XP-CLR-based selective sweep analyses, we uncovered key genomic regions associated with cold tolerance and fruit size. These findings provide novel insights into the molecular mechanisms underlying environmental adaptation and fruit development in wax apple, and lay a foundation for molecular breeding and trait improvement.

## 2 Materials and methods

### 2.1 Data Sources

The diploid reference genome of *S. samarangense* (wax apple) used in this study was obtained from the genome assembly published by Zhang et al (Zhang et al., 2024). Whole-genome resequencing (WGS) data and RNA-Seq data for multiple wax apple accessions (**Supplementary Table S1**) were retrieved from the National Genomics Data Center (NGDC; https://ngdc.cncb.ac.cn/). Specifically, the WGS dataset (accession number: PRJCA011699) (Wei et al., 2023) was used for pan-genome construction, variant calling, and population structure analysis, while the RNA-Seq dataset (accession number: PRJCA020470) was utilized to support novel gene prediction, gene expression analysis, and functional annotation.

### 2.2 Cold tolerance assessment

Cold tolerance was evaluated based on relative electrolyte conductivity (REC) and malondialdehyde (MDA) content. Plants were subjected to low-temperature treatments in a GXZ-0288 illuminated growth chamber (Ningbo Jiangnan Instrument Factory, Ningbo, China), with temperature fluctuations controlled within ±1°C. Under dark conditions, plants were exposed to stepwise temperature gradients of 7°C, 4°C, 1°C, and -2°C, with each treatment lasting 12 days. One plant was used per treatment, and each treatment included three biological replicates. Immediately after treatment, fully expanded mature leaves were collected and divided into two portions for REC and MDA measurements.

For REC determination, four mature leaves were randomly collected from different orientations of each plant, washed thoroughly, and rinsed with ultrapure water. After blotting dry, leaf veins were removed and leaf tissues were cut into approximately 0.5 cm^2^ fragments and homogenized. A total of 1 g of leaf tissue was transferred into an Erlenmeyer flask containing 20 mL of ultrapure water, followed by vacuum infiltration for 15 min. Samples were incubated at room temperature for 4 h, and the initial electrical conductivity (R1) was measured using a DDS-11A precision digital conductivity meter. The samples were then heated in boiling water for 15 min, cooled to room temperature, and the final conductivity (R2) was measured. REC was calculated as R1/R2 × 100%.

MDA content was quantified using a commercial assay kit (Suzhou Michy Biomedical Technology Co., Ltd., Suzhou, China). Briefly, 0.3 g of fresh leaf tissue was homogenized in 1 mL of extraction buffer at room temperature and centrifuged at 10,000 rpm for 10 min. An aliquot of 100 μL supernatant was mixed with 300 μL reaction reagent and incubated in a 92°C water bath for 30 min. After cooling, the mixture was centrifuged at 12,000 rpm for 10 min at 25°C. Absorbance of the supernatant was measured at 532 nm and 600 nm using a spectrophotometer, and ΔA was calculated as A532 − A600. MDA content (nmol/g fresh weight) was calculated as 25.58 × (ΔA + 0.0076)/W, where W represents the fresh weight of the sample.

### 2.3 Pangenome construction

To construct the *S. samarangense* pangenome, we utilized WGS data from 27 wax apple accessions. Raw sequencing reads were first subjected to quality control using fastp (v0.23.4) (Chen et al., 2018) with default parameters. Cleaned reads were then aligned to the diploid reference genome using BWA (Li and Durbin, 2009), and the resulting alignments were sorted using the sort function in SAMtools (v1.13) (Danecek et al., 2021). Unmapped reads were extracted using the fastq module of SAMtools for subsequent *de novo* assembly. The unmapped reads from each accession were independently assembled using MaSuRCA (v3.2.1) (Zimin et al., 2013) to generate contigs. All contigs from the 27 samples were merged, and redundant sequences were removed using CD-HIT-EST (v4.8.1) (Fu et al., 2012) with default settings. To further eliminate redundancy, an all-vs-all sequence comparison was performed using BLASTN (E-value ≤ 1e-5) and nucmer (Kurtz et al., 2004); sequences with over 90% identity and 90% coverage to the reference genome were removed. The resulting non-redundant novel sequences were then filtered against the NCBI NT database using BLASTN, and sequences that came from non-Viridiplantae organisms (e.g., archaea, viruses, bacteria, fungi, and animals) were discarded. To exclude organellar sequences, we downloaded the mitochondrial and chloroplast genome sequences of *S. samarangense* from NCBI (**Supplementary Table 2**) and used BLASTN to remove any contigs of mitochondrial or chloroplast origin. Finally, the filtered novel sequences were once again aligned to the reference genome using BLASTN, and any remaining sequences with high similarity to the reference were excluded. The final set of unique novel sequences was then merged with the reference genome to form the *S. samarangense* pangenome.

### 2.4 Gene structure annotation

To annotate the novel sequences of the *S. samarangense* pangenome, transcriptome data from 27 wax apple samples were first downloaded from the NCBI database. Transcriptome assembly for each sample was performed using SOAPdenovo-Trans (v1.0.5) (Xie et al., 2014) and Trinity (v2.15.1) (Grabherr et al., 2011). The assembled transcriptomes from all samples were then merged and redundant sequences were removed using CD-HIT-EST, producing a non-redundant transcript set to serve as transcriptomic evidence for gene annotation. To train a homologous protein model for gene prediction, we used AUGUSTUS (v3.5.0) (Stanke et al., 2006), which was trained on protein sequences from the reference genome. To minimize the interference of repetitive elements during gene structure prediction, we identified repetitive sequences in the novel genomic regions using three complementary approaches. First, RepeatMasker (v4.1.7) (Chen, 2004) was applied with the RepBase transposable element library (v17.01, http://www.girinst.org/repbase) to detect known transposable elements. Second, a *de novo* repeat library was constructed using RepeatModeler (v2.0.5) (Flynn et al., 2020), and repetitive elements were annotated using RepeatMasker. Third, tandem repeats were identified using Tandem Repeats Finder (TRF) (v4.09) (Benson, 1999). The outputs from all three methods were merged, and the identified repeats were masked in the novel sequences. Gene structure prediction was then performed on the repeat-masked novel sequences using MAKER2 (v2.31.10) (Cantalapiedra et al., 2021). Protein-coding genes with amino acid sequences longer than 50 residues and annotation edit distance (AED) ≤ 0.5 were retained as high-confidence gene models.

For functional annotation, eggNOG-mapper (v2.1.2) (Cantalapiedra et al., 2021) was used to assign Gene Ontology (GO) terms to each gene. KOBAS (v2.0) (Xie et al., 2011) was employed to annotate KEGG Orthology (KO) terms and assign genes to KEGG pathways. Additionally, HMMER (hmmsearch, v3.4) (Eddy, 2023) was used to annotate Pfam domains in the protein-coding genes. Gene enrichment analyses were conducted using the clusterProfiler R package (Wu et al., 2021).

### 2.5 Population analysis based on PAV

To investigate population structure based on gene PAV, WGS reads were first remapped to the constructed wax apple pangenome using BWA. Gene-level PAVs were identified using SGSGeneLoss (v0.1) (Golicz et al., 2015), with parameters set to minCov = 2 and lostCutoff = 0.2, meaning a gene was considered present if at least two reads covered more than 20% of its exon regions. A binary PAV matrix was then generated and used for multidimensional analyses of population genetic structure. Principal component analysis (PCA) was conducted using the vegan R package (Oksanen et al., 2015). A maximum likelihood phylogenetic tree was constructed using IQ-TREE (v2.3.3) (Minh et al., 2020) with the options -st BIN -alrt 1000, which are optimized for binary input data. Additionally, population structure was inferred using the binary matrix in STRUCTURE (v2.3.4) (Hubisz et al., 2009).

Based on the PAV matrix, genes in the pangenome were categorized as core genes (present in all 27 accessions), dispensable genes (present in 1–26 accessions), and private genes (present in only one accession). Gene frequencies were calculated to assess potential selection pressures acting on PAVs during population divergence. Differences in gene frequencies between groups were evaluated using Fisher’s exact test, with p-values adjusted by the Benjamini-Hochberg (BH) method. Statistical significance was defined as a false discovery rate (FDR) < 0.001 and |log_2_ fold change| > 1.

### 2.6 Population genetic analysis based on SNPs

SNP calling was performed using GATK (v4.6.1) (McKenna et al., 2010) with the following pipeline: First, potential PCR duplicates were identified and removed using the “MarkDuplicates” tool. Variant calling was then conducted using HaplotypeCaller to generate GVCF files. The raw SNPs were filtered using VariantFiltration with parameters “FS>30.0 || QD<2.0” to remove low-quality variants. We subsequently applied SelectVariants with parameters “-exclude-filtered true --restrict-alleles-to BIALLELIC” to retain only high-quality biallelic SNPs. Further filtering was performed using VCFtools (v0.1.16) (Danecek et al., 2011) with parameters “--max-missing 0.7 --maf 0.5” to remove SNPs with >30% missing data across populations and minor allele frequency (MAF) <5%.

Population structure was analyzed using ADMIXTURE (v1.3.0) (Alexander et al., 2009) with default parameters. For phylogenetic analysis, SNP sequences were extracted and used to construct a maximum-likelihood tree with CASTER-site (v1.19.1.4) (Zhang et al., 2023) under default parameters, with subsequent visualization performed using iTOL (https://itol.embl.de/upload.cgi).

### 2.7 Detection of selective signatures

We employed the XP-CLR (Python version, https://github.com/hardingnj/xpclr) (Chen et al., 2010) to identify selective sweeps between different wax apple populations. The genome was divided into non-overlapping 10 kb windows, and the average XP-CLR likelihood score was calculated for each window. Windows with scores in the top 5% genome-wide were considered strong selective signals. Adjacent windows or those separated by a single window (within the top 10% XP-CLR scores) were merged, with the maximum average score assigned to the new merged region. For candidate genes and their ±2 kb flanking regions, we evaluated population differentiation using *F*_ST_ values computed by VCFtools.

### 2.8 SV calling and filtering

We performed SV detection using Delly (v1.2.6) (Rausch et al., 2012) with the “call” module for individual samples, followed by merging results using Delly’s “merge” tool. The merged SVs were converted to VCF format using bcftools (v1.18) (Danecek et al., 2021). Subsequent filtering was conducted with VCFtools using parameters “--max-missing 0.5 --maf 0.01” to retain variants with ≤50% missing data across samples and minor allele frequency (MAF) ≥1%. Only variants exceeding 50 bp in length were classified as SVs for downstream analyses.

### 2.9 SV hotspot identification

SV hotspots were identified following the method described by Qin et al (Qin et al., 2021). Briefly, we calculated SV breakpoint distributions across the genome using 200 kb sliding windows with 100 kb steps along each chromosome. Windows were ranked in descending order based on SV counts, with the top 5% of windows containing the highest SV breakpoint frequencies designated as hotspots. Adjacent hotspot windows were subsequently merged into contiguous “hotspot regions”.

### 2.10 Genome-wide genetic differentiation analysis

Genome-wide population differentiation was assessed by calculating fixation index (*F*_ST_) and nucleotide diversity (Pi) values for both SVs and SNPs using VCFtools. The genome was partitioned into non-overlapping 100 kb windows, with average *F*_ST_ and Pi values computed for each window. Regions with *F*_ST_ > 0.15 were considered to exhibit significant genetic differentiation between wax apple populations.

### 2.11 PCR and quantitative real-time PCR analysis

Total RNA was extracted using the UPure Tissue RNA Kit (Biokeystone, China), and RNA quality and concentration were assessed by spectrophotometry and agarose gel electrophoresis. First-strand cDNA was synthesized from 1 μg of total RNA using a reverse transcription kit according to the manufacturer’s instructions.

Conventional PCR was performed to validate the presence and absence of the pangenome novel genes. Primers were designed using Primer3 software (**Table 1**). PCR reactions were carried out in a total volume of 20 μL containing cDNA template, gene-specific primers, and PCR master mix. The amplification program consisted of an initial denaturation at 95°C for 3 min, followed by 35 cycles of denaturation at 95°C for 30 s, annealing at 60°C for 30 s, and extension at 72°C for 30 s, with a final extension at 72°C for 5 min. PCR products were analyzed by 1.5% agarose gel electrophoresis.

**Table 1:** Primers used in this study.

Gene	Forward (5’-3’)	Reverse (5’-3’)
evm.TU.group11.2151	TGTGGACTGAGCATCTTCACT	TCAAGGCCTCCGAAGACTTCT
novel_gene00049	GTCAGCATGTATGGGCTAACTC	ATGCTTCACTCTACTCCATATT
evm.TU.group6.4267	CAGAACCAGGGGCTCGAAAAAG	ATGGAGGCCTTCTCTGTCTCTC
evm.TU.group5.517	TGGCTACAACTTTAGGAGTCTT	TCAGATCTTCTCCATCTTCAG
evm.TU.group6.4268	TGAAGATGGAATCGCTCTCGT	CTAGGGTCTCAGGAAGGCCAATC
evm.TU.group7.4063	TACAGGAGAAGAACTAAGACCA	ATGACCGAACACATCAATGCAG

Quantitative real-time PCR (qRT-PCR) was conducted using a SYBR Green–based detection system on a real-time PCR instrument. Total RNA was reverse-transcribed into cDNA with TUREscript 1st Stand cDNA SYNTHESIS Kit (Aidlab, China). Gene-specific primers were designed using Primer3 software (F: TGTGACCGACTCTTGTTC, R: CAGCAGCATCAACTCTTC). Each reaction was performed in a 20 μL volume containing diluted cDNA, gene-specific primers, and SYBR Green PCR master mix. Melting curve analysis was performed to confirm amplification specificity. Relative gene expression levels were calculated using the 2^-ΔΔCt^ method, with an internal reference gene (SsSKIP16, F: GGAACCTCCACTCTGTTCCA, R: AGTCGTAGGGCATTCCATTG) used for normalization.

## 3 Results

### 3.1 Construction of the wax apple pangenome

**Figure 1:**
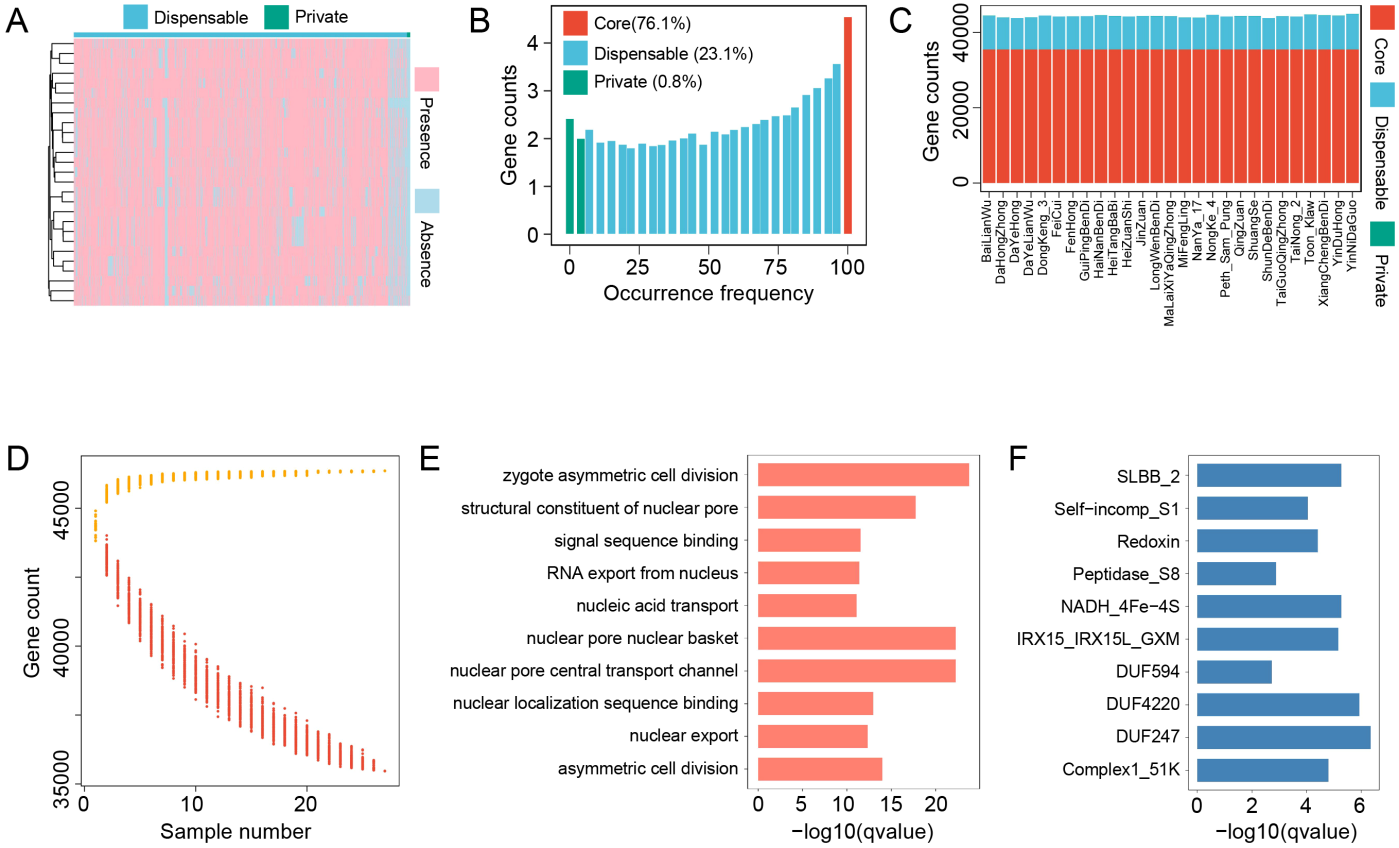
Construction of the wax apple pangenome. **(A)** PAV heatmap of dispensable and private genes. Rows represent 27 samples along with a hierarchical clustering tree, and columns represent genes. **(B)** Frequency distribution of core, dispensable, and private genes. The x-axis indicates gene occurrence frequency, while the y-axis shows the number of genes at each frequency. Different colors represent different PAV types. **(C)** Gene counts of different PAV categories across all samples. **(D)** Curve fitting of pangenome gene number and core gene number in the pangenome as the sample size increases. **(E)** GO enrichment analysis of dispensable genes. **(F)** PFAM domain enrichment analysis of dispensable genes.

In this study, we constructed the *S. samarangense* pangenome using WGS data from 27 cultivars. In addition to the reference genome, 69 Mb of novel sequences were assembled, comprising 41,045 contigs. Gene structure annotation identified 707 novel protein-coding genes within these sequences. Based on the PAV identification of all genes in the pangenome, a total of 46,621 genes were categorized into 35,468 core genes, 10,789 dispensable genes, and 364 private genes (**Figures 1A, B**). We examined the PAV profiles across all samples (**Figure 1C**), finding that the number of genes present in each accession ranged from 43,817 to 44,906. Core genes accounted for approximately 80% of the genes in each individual, whereas private genes represented only a very small proportion, suggesting their highly restricted distribution and potential cultivar specificity. As the number of samples increased, the total number of pangenome genes gradually rose and approached saturation, indicating that the constructed pangenome captures the genetic diversity of *S. samarangense*. In contrast, the number of core genes steadily declined with increasing sample size, implying differences in the core gene repertoire across populations (**Figure 1D**). Furthermore, PCR amplification of randomly selected sequences confirmed the reliability of the predicted PAVs in this study (**Supplementary Figure S1**).

Given the widespread PAV of dispensable genes, which may underlie phenotypic differences among populations, we performed functional enrichment analysis on these genes. GO enrichment revealed significant associations with terms related to asymmetric cell division (e.g., zygote asymmetric cell division, asymmetric cell division), nuclear pore complex components (e.g., nuclear pore central transport channel, nuclear pore nuclear basket), and nucleic acid transport (e.g., RNA export from nucleus, nucleic acid transport) (**Figure 1E**, **Supplementary Table 3**). Pfam domain enrichment showed overrepresentation of domains such as Complex1_51K, Peptidase_S8, IRX15_IRX15L_GXM, DUF247, and Self-incomp_S1 (**Figure 1F**, **Supplementary Table 4**).

### 3.2 Population structure and phylogenetic analysis of the wax apple

**Figure 2:**
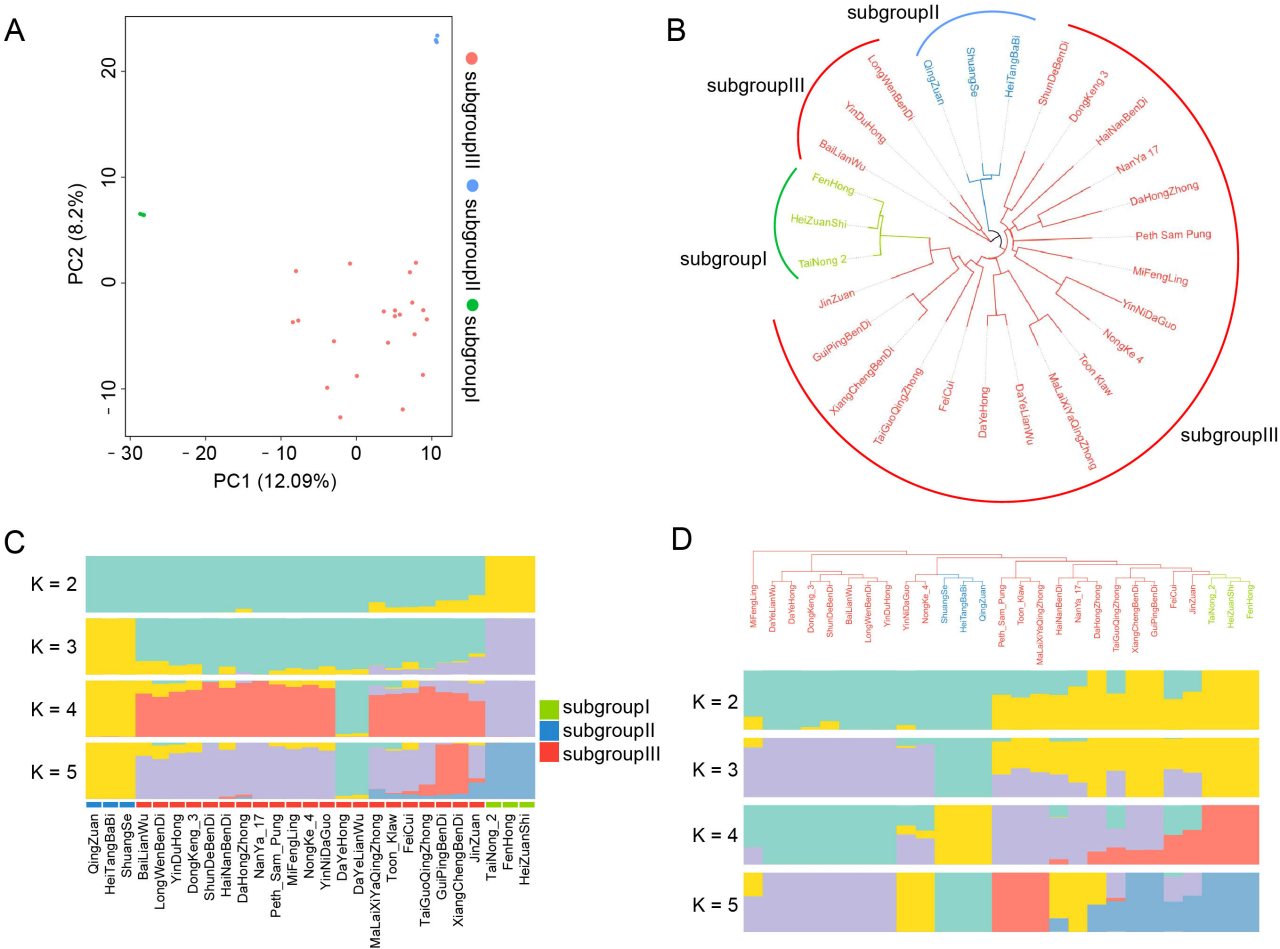
Phylogenetic and population structure analysis of wax apple based on PAV and SNP. **(A)** PCA based on PAVs. Each dot represents a sample, and colors indicate groupings derived from PCA. **(B)** Phylogenetic tree constructed using the binary PAV matrix. **(C)** Population structure analysis based on PAVs, showing inferred clusters under K = 2, 3, 4, and 5. **(D)** Phylogenetic tree and population structure analysis based on SNPs.

To investigate the genetic structure of *S. samarangense* cultivars, we first performed PCA based on the binary PAV matrix. The results revealed a clear tri-group differentiation: Subgroup I comprised FenHong, HeiZuanShi, and TaiNong_2; Subgroup II included HeiTangBaBi, QingZuan, and ShuangSe; while the remaining 21 cultivars clustered into Subgroup III (**Figure 2A**). A phylogenetic tree constructed from the binary PAV matrix further supported this population structure: members within Subgroups I and II formed well-supported monophyletic clades with short branch lengths, indicating close genetic relationships within each subgroup (**Figure 2B**). Population structure analysis using STRUCTURE also supported the three-group division, with the optimal K value (K = 3) corresponding to the clustering observed in PCA and phylogenetic analysis. Notably, the two smaller subgroups (each with three cultivars) were clearly differentiated from the main cultivar group (21 accessions), suggesting that these accessions may have undergone distinct domestication trajectories or breeding selection (**Figure 2C**).

However, population structure analysis based on SNPs revealed a partially different pattern (**Figure 2D**). While HeiTangBaBi, QingZuan, and ShuangSe remained a separate cluster, the subgroup containing FenHong, HeiZuanShi, and TaiNong_2 did not form an independent group and instead clustered with the main cultivars. Interestingly, the SNP-based phylogenetic tree still maintained a three-clade structure. This discrepancy may be attributed to the different temporal sensitivities of molecular markers: PAVs may retain deeper historical signals, such as ancient lineage divergence or early selection events, whereas SNPs may better capture recent gene flow and convergence driven by cultivation practices.

### 3.3 Population selection analysis in wax apple

As a key tropical fruit crop, *S. samarangense* exhibits substantial phenotypic diversity across cultivars, particularly in fruit size and environmental adaptability. For example, cultivars such as HeiTangBaBi and YinNiDaGuo produce notably larger fruits, while DaYeHong and DongKeng 3 demonstrate enhanced cold tolerance under low-temperature conditions (**Supplementary Table 5**, **Supplementary Figures S2**, **S3**). To investigate the genetic basis of these phenotypic differences, we first analyzed gene frequency based on the PAV across distinct population groups. A total of 212 genes showed significantly higher presence frequencies in cold-tolerant varieties, while 311 genes were showed significantly higher presence frequencies in large-fruited cultivars (**Supplementary Tables 6**, **7**). Functional annotations revealed that some of these genes, which are under PAV selection, are associated with phenotype-relevant functions. For example, *evm.TU.group2.3467* exhibits a significantly higher frequency in cold-tolerant cultivars and is annotated with a PPR domain in the Pfam database, which is associated with cold resistance. Similarly, *evm.TU.group5.517* shows a significantly higher frequency in large-fruited cultivars and contains a P450 domain, which is linked to fruit size. Importantly, several key genes were located on novel sequences uncovered by pangenome assembly. For instance, *novel_gene00318*, annotated with a PFAM domain LRR_1, is potentially involved in disease resistance. These findings underscore the power of the pangenome in capturing gene variants missed by the reference genome.

**Figure 3:**
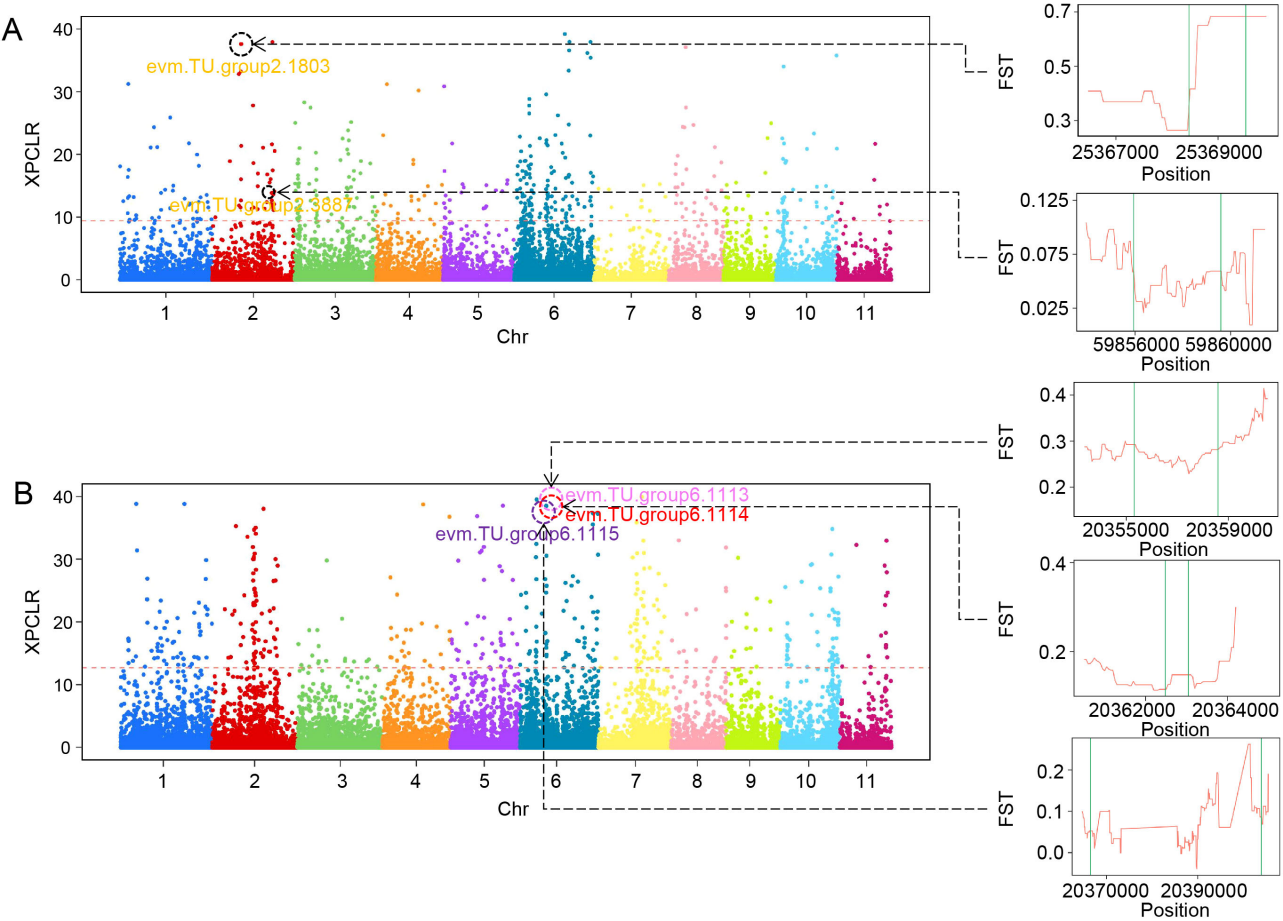
Selective sweeps in wax apple. **(A)** Genome-wide distribution of XP-CLR likelihood scores between cold-tolerant (most cold-resistant and cold-resistant) and non-cold-tolerant populations. The line chart on the right represents *F*_ST_ values across the gene regions and 2 kb upstream and downstream of *evm.TU.group2.1803* and *evm.TU.group2.3887*. **(B)** Genome-wide distribution of XP-CLR likelihood scores between large-fruit (single fruit weight ≥100g) and small-fruit (single fruit weight ≤70g) populations. The line chart on the right represents *F*_ST_ values across the gene region and 2 kb upstream and downstream of *evm.TU.group6.1113*, *evm.TU.group6.1114*, and *evm.TU.group6.1115*.

In addition, we performed XP-CLR analysis to identify genomic regions under selection between contrasting groups for cold tolerance and fruit size. We identified ~8.7 Mb and ~9.2 Mb of regions with significant selection signals between cold-tolerant vs. cold-sensitive (**Figure 3A**), and large-fruited vs. small-fruited populations (**Figure 3B**), respectively. In the cold tolerance comparison, 21 genes had selection signals (**Supplementary Table 8**), several of which are functionally relevant to cold response. For example, *evm.TU.group2.1803* was found in a peak region with an XP-CLR likelihood score of 37.6. PFAM annotation identified it as a SAM-dependent methyltransferase involved in quercetin metabolism. Methyltransferases can influence plant development and stress response through epigenetic modifications. Moreover, this gene exhibited a high *F*_ST_ (>0.6) between cold-tolerant and sensitive populations, indicating strong genetic differentiation and suggesting its potential role in cold adaptation. Similarly, *evm.TU.group2.3887*, located in a region with a score of 13.6, belongs to the LRR-RLK gene family, known to play key roles in plant development, hormone signaling, abiotic stress response, and pathogen defense (Shiu and Bleecker, 2001; Xiang et al., 2006; de Lorenzo et al., 2009; Li et al., 2018). For fruit size, 16 genes were located within selected regions (**Supplementary Table 9**). Notably, we identified a homologous gene cluster of Mol-like proteins on chromosome 6, including *evm.TU.group6.1113*, *evm.TU.group6.1114*, and *evm.TU.group6.1115* (**Figure 3B**). This region had an XP-CLR score of 38.5, especially the gene region and upstream and downstream of *evm.TU.group6.1113* showed *F*_ST_ values above 0.15, suggesting that this gene cluster may contribute to the regulation of fruit size. *evm.TU.group6.1115* exhibited a higher number of Mol domains across the three genes in the cluster (**Supplementary Figure S4**), and its expression level was significantly elevated in large-fruited cultivars compared with small-fruited cultivars (**Supplementary Figure S5**), suggesting that this gene may be associated with fruit size. Moreover, SVs were detected within the genomic regions of several genes located in these selection signal intervals. For example, both an SV deletion and an SV duplication were identified within the genomic region of *evm.TU.group2.1803*, while two SV deletions were detected within the regions of *evm.TU.group6.1113* and *evm.TU.group6.1115*, indicating that SVs may be associated with adaptive evolution of genes under selection.

### 3.4 SV landscape in the wax apple population

**Figure 4:**
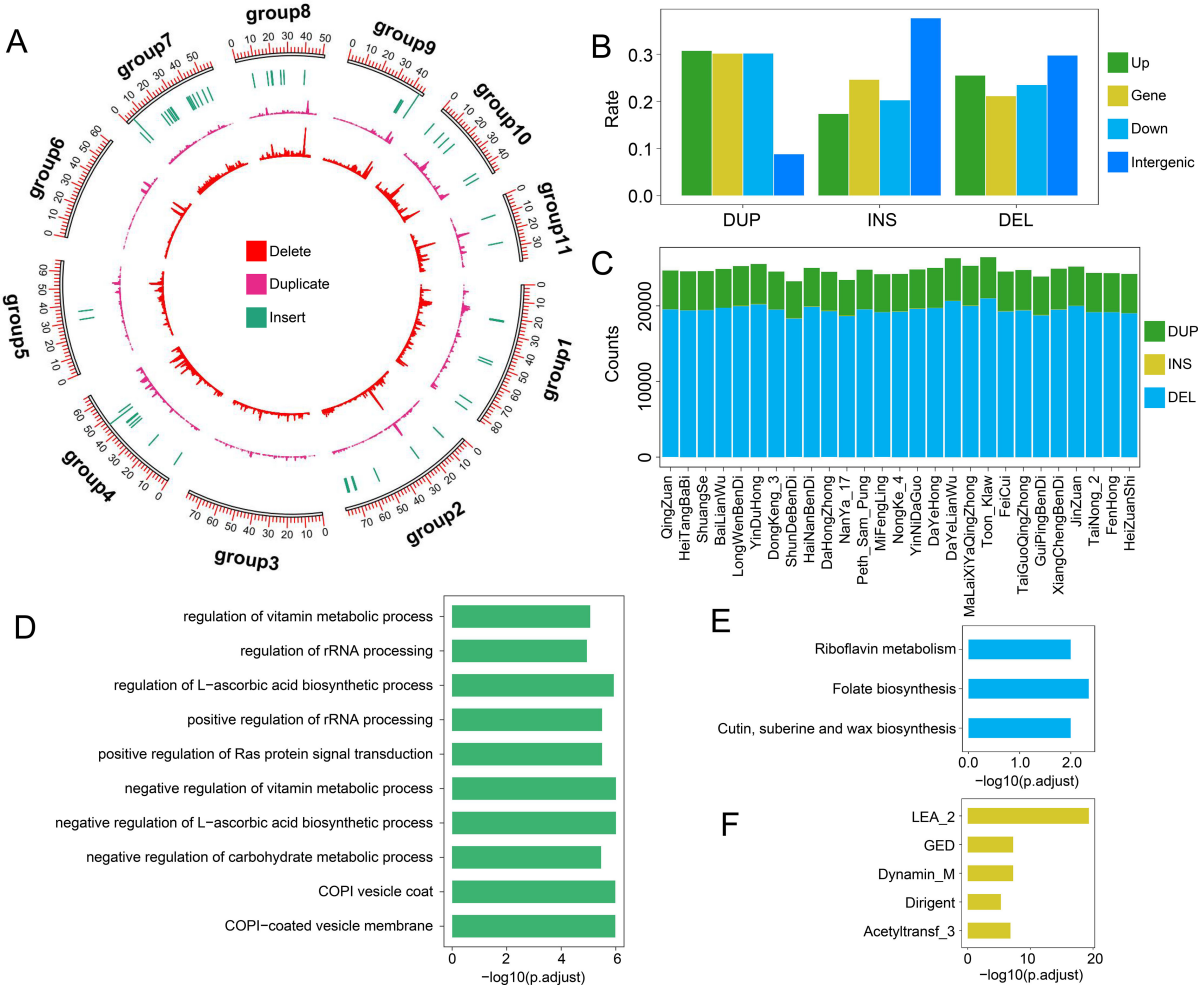
SVs in 27 wax apple samples. **(A)** Distribution of SV counts across all samples. **(B)** Genomic distribution of three SV types (deletion, duplication, and insertion) across gene regions, 2 kb upstream/downstream, and intergenic regions. **(C)** Chromosomal distribution of deletions, duplications, and insertions SVs across the 11 wax apple chromosomes. **(D–F)** GO **(D)**, KEGG **(E)**, and PFAM **(F)** enrichment analyses of genes located within SV hotspot regions, respectively.

SVs play crucial roles in plant genome evolution, agronomic trait regulation, and environmental adaptation. In this study, we comprehensively identified SVs across the wax apple population. A total of 44,657 SVs were detected, including 9,999 duplications, 34,593 deletions, and 65 insertions. The number of SVs per sample ranged from 23,252 to 26,411, with deletions accounting for approximately 78.35%–79.6% of all SVs. Insertions were rare, with fewer than 50 per sample (**Figure 4A**). Regarding their genomic locations, most duplication SVs were found within gene regions or in the gene upstream and downstream regions of genes. Deletion SVs were distributed more evenly among gene regions, upstream/downstream regions, and intergenic regions. Notably, 31.1% of insertion SVs were located in intergenic regions (**Figure 4B**). As SVs were not uniformly distributed across the genome (**Figure 4C**), we performed a hotspot analysis to identify regions with high SV density. A total of 67 SV hotspots were identified. Although these hotspots accounted for only 6% of the genome (~41 Mb), they harbored 87.7% of all SVs (39,063), suggesting their potential importance in genome structure and function.

To investigate the functional implications of these SV-enriched regions, we performed enrichment analysis of genes located within the hotspots. The results showed that these genes were significantly enriched in pathways related to secondary metabolism, particularly those involving vitamin biosynthesis and metabolism. GO enrichment analysis highlighted terms such as COPI-coated vesicle membrane, COPI vesicle coat, negative regulation of carbohydrate metabolic process, negative regulation of L-ascorbic acid biosynthetic process, positive regulation of Ras protein signal transduction, and regulation of rRNA processing (**Figure 4D**). KEGG analysis revealed enrichment in folate biosynthesis, riboflavin metabolism, and cutin, suberine and wax biosynthesis pathways (**Figure 4E**). PFAM domain enrichment indicated a prevalence of domains such as LEA_2, GED, Dynamin_M, Dirigent, and Acetyltransf_3 (**Figure 4F**). These findings provide new insights into the potential genetic basis underlying fruit quality and stress tolerance in wax apple.

### 3.5 SV-driven population differentiation analyses

**Figure 5:**
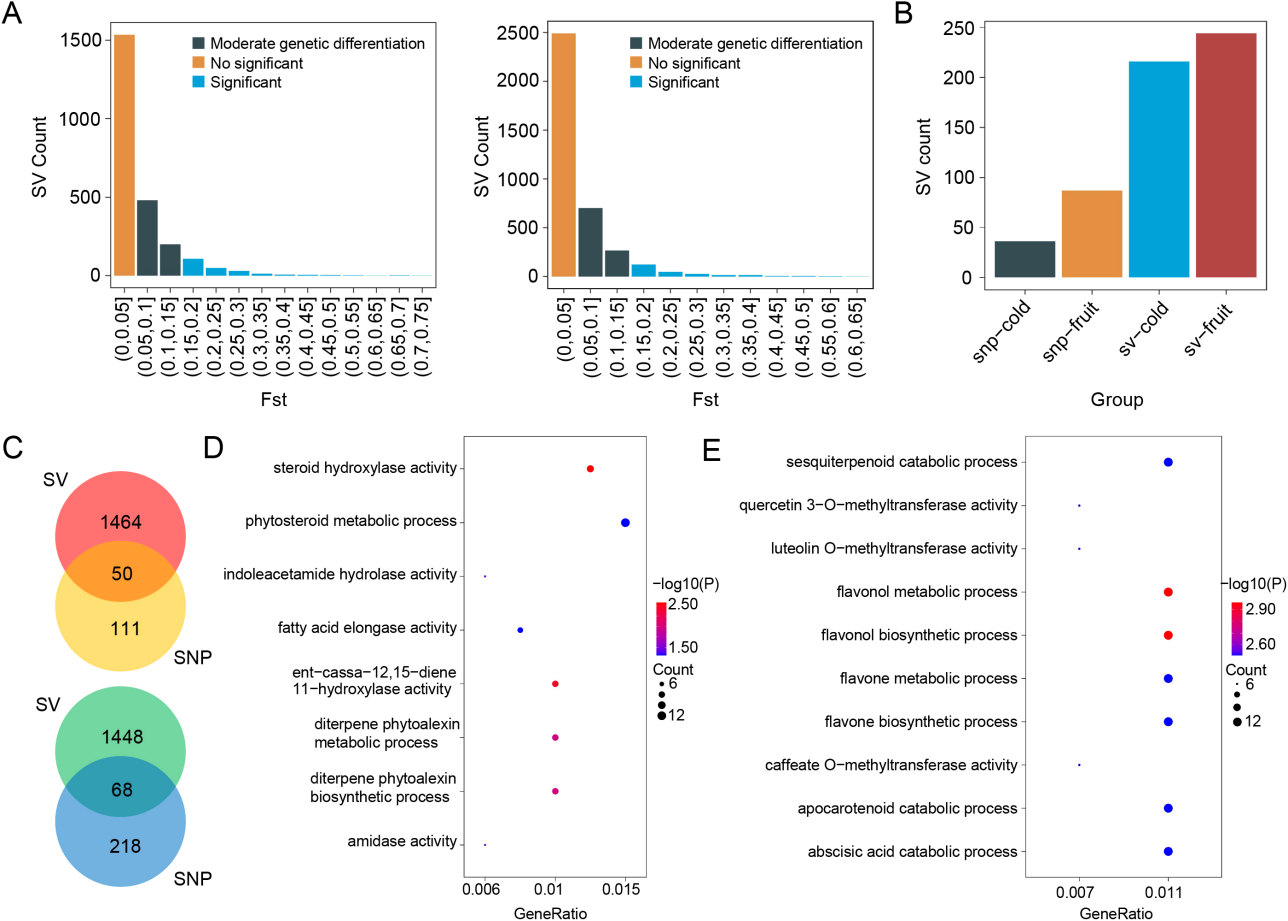
Population differentiation based on SVs. **(A)** Distribution of *F*_ST_ values between groups with differing cold tolerance and fruit size. When *F*_ST_ < 0.05, genetic differentiation between groups is minimal and considered non-significant. *F*_ST_ values between 0.05 and 0.15 indicate moderate genetic differentiation, while *F*_ST_ ≥ 0.15 suggests significant genetic differentiation. **(B)** Comparison of the number of significantly differentiated genomic windows (window size = 100,000 bp) identified using SNPs and SVs. **(C)** Comparison of the number of genes located within significantly differentiated regions identified using SNPs and SVs. **(D)** GO enrichment analysis of genes located in significantly differentiated regions between groups with differing cold tolerance. **(E)** GO enrichment analysis of genes located in significantly differentiated regions between groups with differing fruit size.

SV as a major source of genomic variation may play a crucial role in adaptive evolution and phenotypic diversity. However, the contribution of SVs to population differentiation and key traits such as cold tolerance and fruit size in wax apple remains unclear. In this study, we calculated SV-based *F*_ST_ values between populations with different levels of cold tolerance and fruit size, identifying 216 and 244 significantly differentiated regions, respectively (**Figure 5A**). These regions contained 1,514 and 1,516 genes, respectively. Compared with SNP-based *F*_ST_ analysis under the same parameters, SV-based *F*_ST_ identified a larger number of significantly differentiated genomic regions (**Figure 5B**). Moreover, the number of genes associated with SVs exceeded those associated with SNPs. This phenomenon is likely because SVs span longer sequences than SNPs, which involve only a single base change. The overlap between SV-associated and SNP-associated genes was minimal (**Figure 5C**), suggesting that these two types of variants may influence trait differentiation through distinct genetic mechanisms. To further explore the functions of genes located in SV-driven differentiated regions, we performed gene enrichment analysis. In the cold-tolerant and cold-sensitive populations, genes within significantly differentiated regions were enriched in GO terms related to plant growth, development, and environmental adaptation, including amidase activity, diterpene phytoalexin biosynthetic process, diterpene phytoalexin metabolic process, ent-cassa-12,15-diene 11-hydroxylase activity, fatty acid elongase activity, indoleacetamide hydrolase activity, phytosteroid metabolic process, and steroid hydroxylase activity (**Figure 5D**).

## 4 Discussion

Compared to a single reference genome, a pangenome provides a more comprehensive representation of the genetic diversity within a species. In recent years, significant progress has been made in pangenome research across various plant species. For example, the tomato pan-genome (1,179 Mb) revealed 351 Mb of novel sequences and 4,873 new genes (Gao et al., 2019); the sunflower pangenome identified 17,061 new genes (Hübner et al., 2019); and analysis of 3,010 accessions of Asian cultivated rice uncovered 268 Mb of non-redundant novel sequences containing 12,465 new genes (Wang et al., 2018). In this study, we assembled a pangenome based on 27 diploid *S. samarangense* accessions and assembled 69 Mb of novel sequences and 707 new genes. Compared to other plant species, the scale of novel sequences and gene numbers in the wax apple is relatively limited, which may be attributed to its low genetic diversity. The natural distribution of wax apple is geographically restricted to tropical regions such as Malaysia, Indonesia, the Philippines, and parts of southern China, including Guangdong and Taiwan. This narrow distribution likely constrains the accumulation of genetic variation. From a functional perspective, previous studies have shown that variable genes in pangenomes (i.e., flexible or dispensable genes) are often closely associated with environmental adaptation. For instance, in tomato, variable genes are significantly enriched in defense response pathways (Gao et al., 2019), while in Asian rice, dispensable genes are primarily involved in immune and defense regulation (Wang et al., 2018). In contrast, the variable genes identified in wax apple showed no significant enrichment in pathways related to environmental adaptation or defense, which may reflect its limited distribution and relatively weak adaptability. Interestingly, several unique functional domains were enriched in the Pfam analysis of wax apple genes, including DUF4220, DUF594, Self-incomp_S1, and DUF247. Among them, DUF4220 and DUF594 often occur in tandem and are plant-specific. Although the functions of these domains remain largely unknown, studies suggest they may be involved in sugar metabolism regulation. For example, in rice, the *OsSAC1* encodes a protein containing both DUF4220 and DUF594 domains, and its mutation leads to sugar accumulation in leaves, implying a potential role for these domains in sugar metabolism and distribution (Zhu et al., 2018). Additionally, the Self-incomp_S1 and DUF247 domains are closely linked to plant self-incompatibility (SI). In grasses, DUF247 is widely present in self-incompatible species but often lost or mutated in self-compatible ones (Foote et al., 1994). The absence of Self-incomp_S1 and DUF247 domains in the wax apple genome may be associated with the evolutionary development of its self-compatibility (Manzanares et al., 2016; Herridge et al., 2022). These findings offer insights into the potential roles of dispensable genes in wax apple and provide a foundation for further functional investigations.

Currently, population genetics and genomic studies of wax apple remain in the early exploratory stages. Wax apple exhibits both diploid and tetraploid forms, with a diverse and complex range of chromosome numbers (2n = 33, 42, 44, 66, and 88), suggesting that the species may have undergone polyploidization and/or hybridization events. Although high-quality genomes of diploid and tetraploid wax apple were published in 2023 (Wei et al., 2023) and 2024 (Zhang et al., 2024), respectively, their population structure, origin, and evolutionary history remain largely unresolved. A previous study classified local landraces and cultivated varieties into two major groups (Wei et al., 2023), but only reported population structure results for K = 2. Increasing the K value may reveal finer substructures, indicating that the genetic diversity of wax apple may be more complex than previously recognized. In this study, PAV-based population analysis of 27 local landraces revealed two distinct subgroups. Notably, earlier SNP-based PCA also divided the local landraces into three groups (Wei et al., 2023), consistent with our PAV-based PCA findings. Importantly, both SNP- and PAV-based PCA and phylogenetic analyses support the existence of three major groups: (1) FenHong, HeiZuanShi, and TaiNong_2; (2) HeiTangBaBi, QingZuan, and ShuangSey; and (3) other varieties. These results suggest that current wax apple populations may have originated from three distinct ancestral lineages, pointing to multiple independent domestication or dispersal events. However, research on the origin and evolution of the wax apple is still limited. This hypothesis requires further support from expanded genomic data, population genetics analyses, and paleobotanical evidence. Future studies combining whole-genome resequencing, demographic modeling, and comparative genomics will be critical to unraveling the domestication history and adaptive evolutionary mechanisms of wax apple.

Fruit size and cold tolerance are two key agronomic traits that directly impact the yield and cultivation potential of *S. samarangense*. In this study, we integrated three complementary approaches, including PAV selection, SNP-based XP-CLR selection sweep detection, and SV-based *F*_ST_ population differentiation, to identify candidate genetic loci potentially associated with these traits. PAV selection analysis identified *evm.TU.group5.517*, which exhibits significant frequency differences between different fruit size populations and encodes a protein containing a P450 domain. Previous studies have demonstrated that RNAi-mediated suppression of *P450* alters fruit size in cherry, suggesting a potential conserved role of this gene family in fruit development (Qi et al., 2017). Similarly, *evm.TU.group2.3467*, which shows significant frequency differences between cold-tolerant and cold-sensitive groups, harbors a PPR domain. PPR proteins have been implicated in cold tolerance in rice by regulating mitochondrial superoxide levels (Zu et al., 2023), indicating their likely involvement in cold adaptation in wax apple. XP-CLR analysis further supported the involvement of these genes in adaptive differentiation. For instance, in cold-tolerant populations, the selective region contained *evm.TU.group2.1803*, which is annotated as an S-adenosylmethionine-dependent methyltransferase. A gene with the same domain called *WPEAMT* has been shown to contribute to cold adaptation in wheat (Charron et al., 2002), reinforcing its relevance in low-temperature stress responses. Additionally, the XP-CLR region associated with fruit size contained a *Mol* gene cluster, members of which have been previously linked to fruit development (Feechan et al., 2008; Chen et al., 2014). SV-based *F*_ST_ analysis revealed genomic regions with significant genetic differentiation between groups with contrasting cold tolerance or fruit size. In cold-tolerant vs. cold-sensitive groups, genes within highly differentiated regions were enriched for terms related to diterpene phytoalexin biosynthesis and metabolism. Diterpene phytoalexins are known to act as crucial defense-related secondary metabolites, playing vital roles in plant responses to both biotic and abiotic stresses (Sashankar and Chakraborty, 2023). In contrast, genes showing differentiation between fruit size groups were enriched for terms related to fruit development and hormone signaling. For example, abscisic acid (ABA) is known to regulate ethylene biosynthesis and signaling during fruit ripening, and its crosstalk with other hormones is a major determinant of fruit size and maturation, although the underlying regulatory mechanisms remain largely unclear (Gupta et al., 2022). Moreover, quercetin, a flavonol belonging to the polyphenol family, has been implicated in influencing fruit peel color and ripening in apple, alongside anthocyanin glycosides (Reay and Lancaster, 2001), highlighting its potential role in wax apple fruit quality traits. Collectively, these findings provide valuable genetic insights into the molecular mechanisms underlying fruit development and cold tolerance in wax apple. The candidate genes and pathways identified here offer promising targets for functional validation and molecular breeding. However, the roles of these candidate genes are currently supported primarily by genomic analysis, and their precise biological functions require further experimental verification through approaches such as gene expression perturbation, transgenic analysis, or genome editing. Future studies integrating functional genomics and multi-omics approaches will be instrumental in constructing regulatory networks of key agronomic traits and accelerating varietal improvement in this species.

## Data Availability

The original contributions presented in the study are included in the article/**Supplementary Material**. Further inquiries can be directed to the corresponding authors.

## Supplementary material

Supplementary Figure 1 Gel electrophoresis validation of pangenome genes.

Supplementary Figure 2 Fruit morphology of wax apple.

Supplementary Figure 3 Cold tolerance assessment of wax apple. **(A)** Phenotypic responses of wax apple leaves under cold stress treatment. **(B)** MDA content in wax apple leaves under cold stress. **(C)** Relative electrolyte conductivity of wax apple leaves under cold stress.

Supplementary Figure 4 Gene structure of fruit size and cold tolerance candidates.

Supplementary Figure 5 The expression level of *evm.TU.group6.1115* in the species with different fruit size. ** represents that there is a significant change in the two samples.

Supplementary Table 1 Information on wax apple accessions and sequencing data.

Supplementary Table 2 Reference organellar genomes of *S. samarangense*.

Supplementary Table 3 GO enrichment analysis of dispensable genes.

Supplementary Table 4 Pfam domain enrichment analysis of dispensable genes.

Supplementary Table 5 Cultivar names and their key phenotypic traits.

Supplementary Table 6 Genes associated with cold tolerance.

Supplementary Table 7 Genes associated with fruit size.

Supplementary Table 8 XP-CLR candidates for cold tolerance.

Supplementary Table 9 XP-CLR candidates for fruit size.
