# Supplementary material for: Pan-genome analysis of wax apple (Syzygium samarangense) and its association with fruit size and cold tolerance
Source: Front Plant Sci. 2026 Feb 3;17:1703197. doi: 10.3389/fpls.2026.1703197 (PMC12960651; doi:10.3389/fpls.2026.1703197)
Supplement: Supplementary Figure 1 — Gel electrophoresis validation of pangenome genes. [file DataSheet1.pdf]

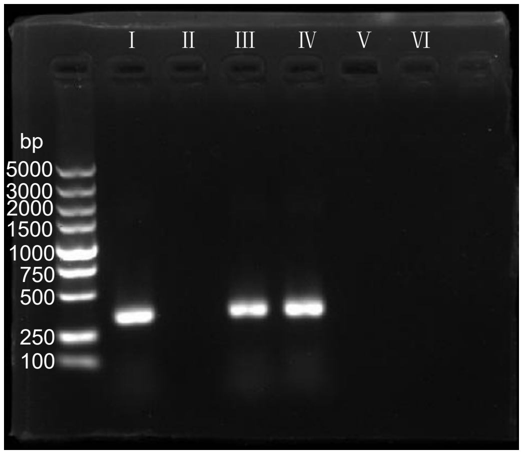

evm.model.group11.2151

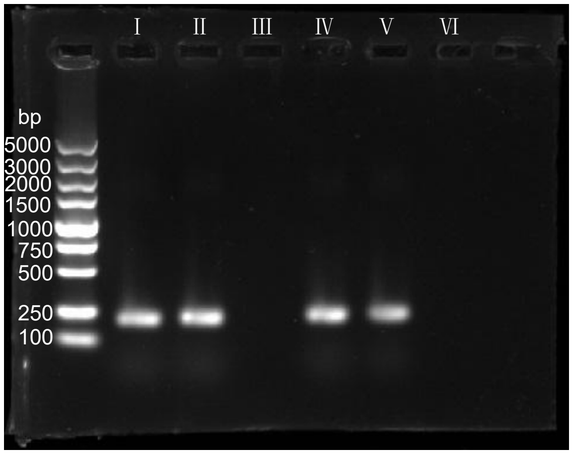

novel_gene00049

Ⅰ HeiTangBaBi
Ⅱ GuiPingBenDi
Ⅲ MiFengLing
Ⅳ DaYeHong
Ⅴ DaHongZhong
Ⅵ TaiGuoQingZhong

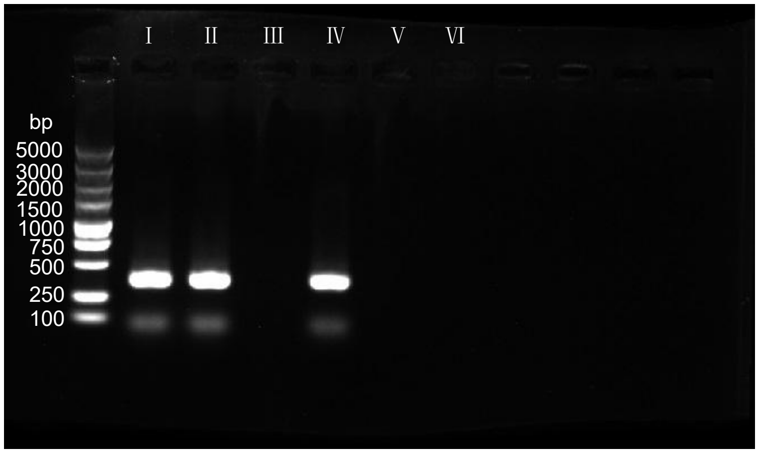

evm.model.group6.4267

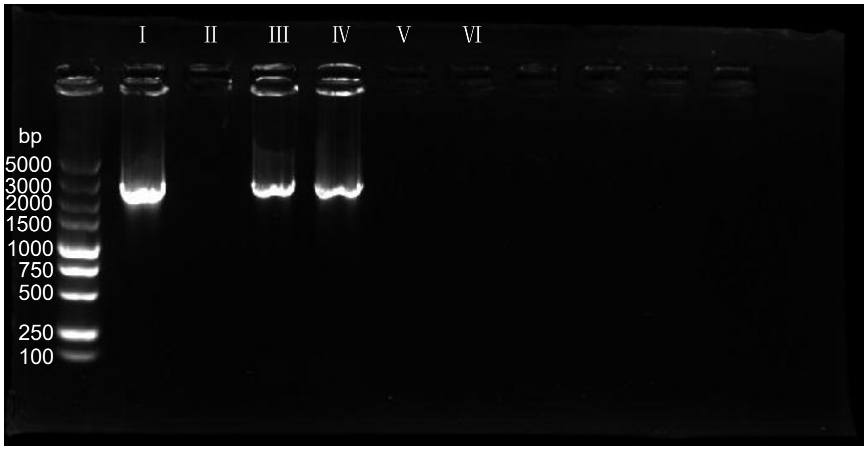

evm.model.group5.517

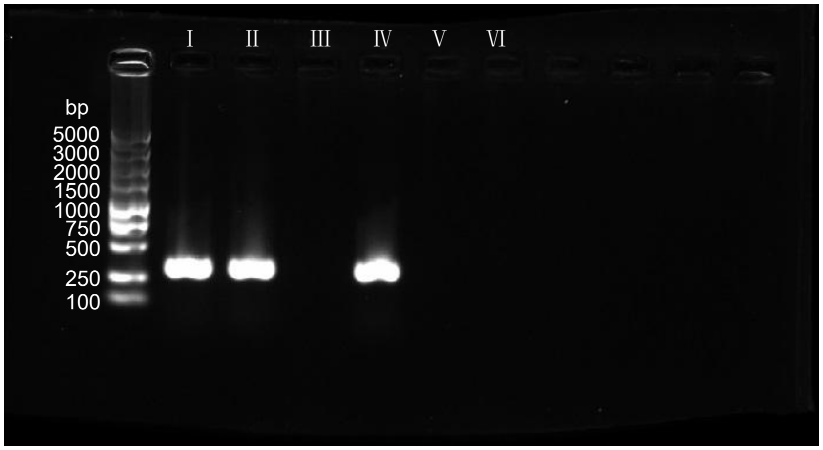

evm.model.group6.4268

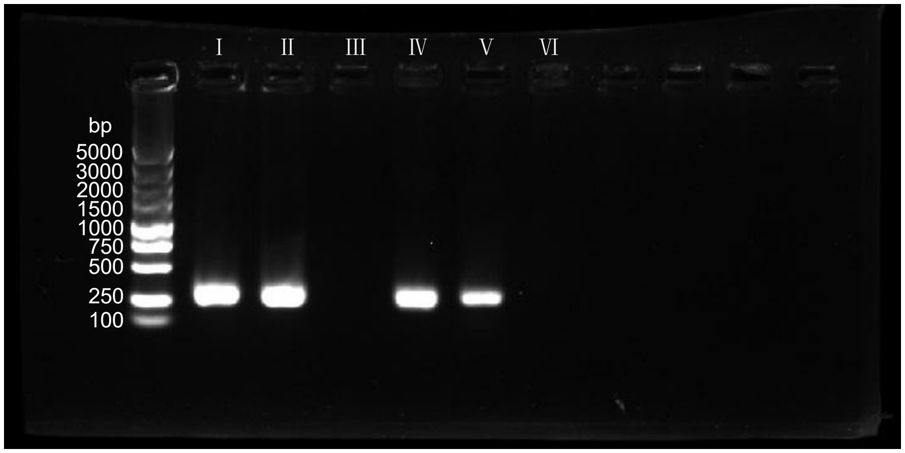

evm.model.group7.4063
