# Supplementary figures and images for: Pan-genome analysis of wax apple (Syzygium samarangense) and its association with fruit size and cold tolerance
Source: Front Plant Sci. 2026 Feb 3;17:1703197. doi: 10.3389/fpls.2026.1703197 (PMC12960651; doi:10.3389/fpls.2026.1703197)

DaYeHong

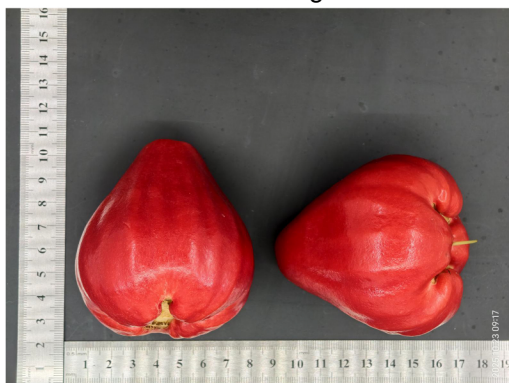

DongKeng\_3

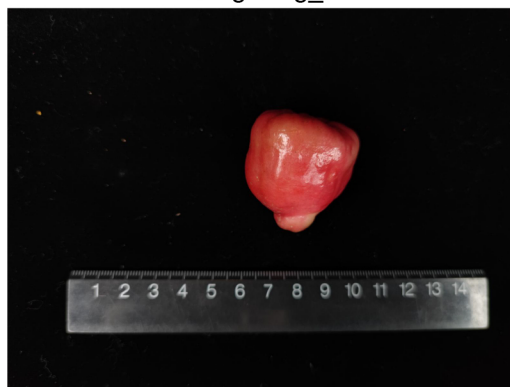

HeiTangBaBi

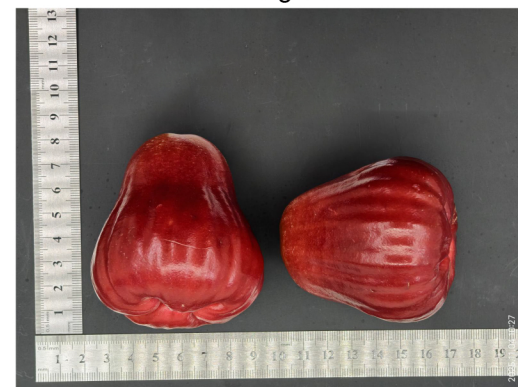

MaLaiXiYaQingZhong

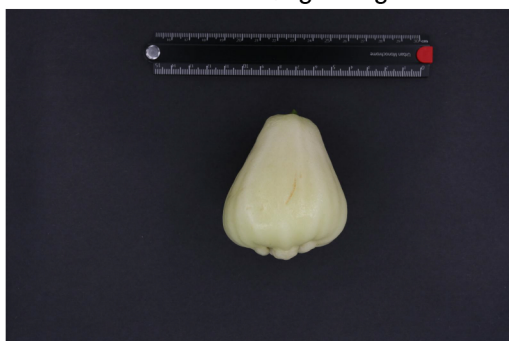

FenHong

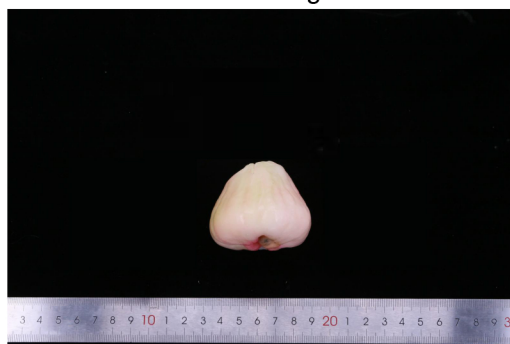

HaiNanBenDi

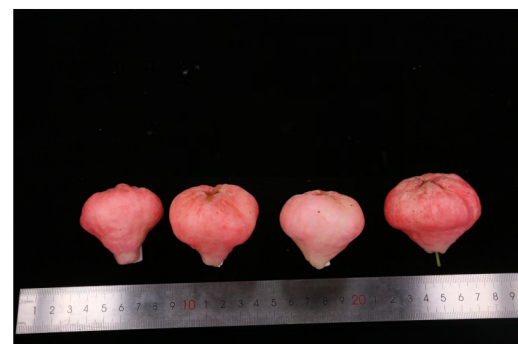

XiangChengBenDi

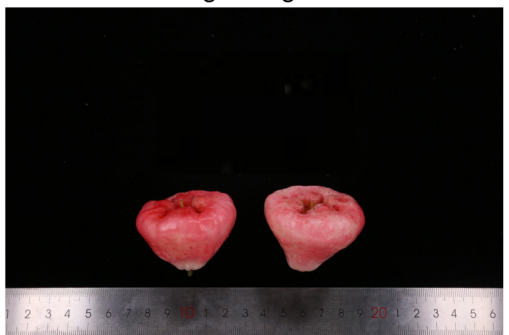

Supplement: Supplementary Figure 2 — Fruit morphology of wax apple. [file DataSheet2.pdf]

A

BaiLianWu

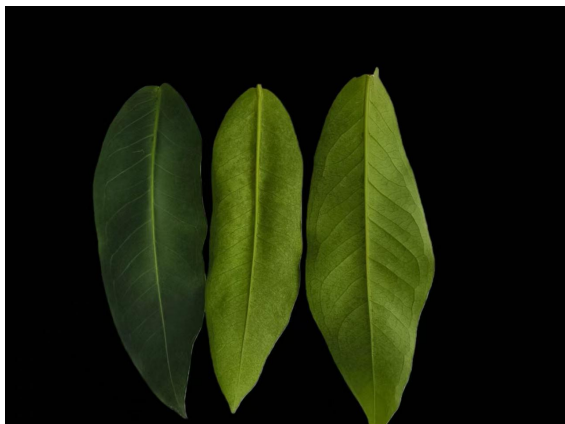

DaYeHong

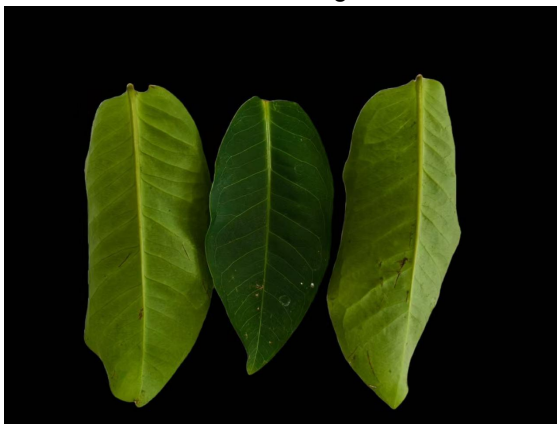

HeiTangBaBi

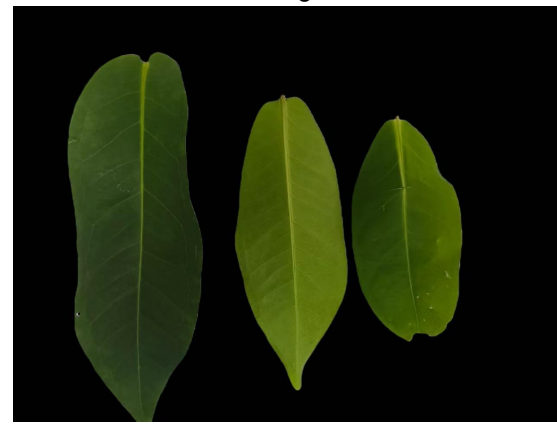

MiFengLing

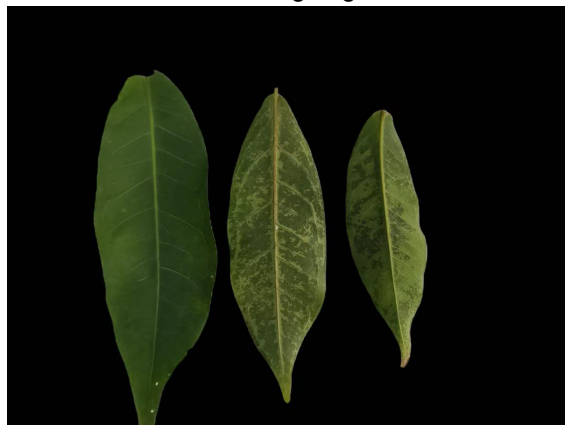

TaiGuoQingZhong

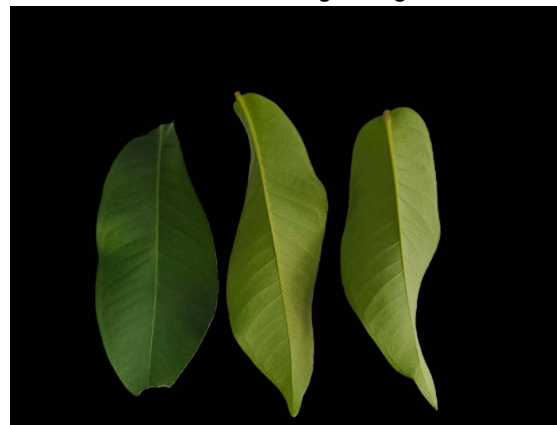

B

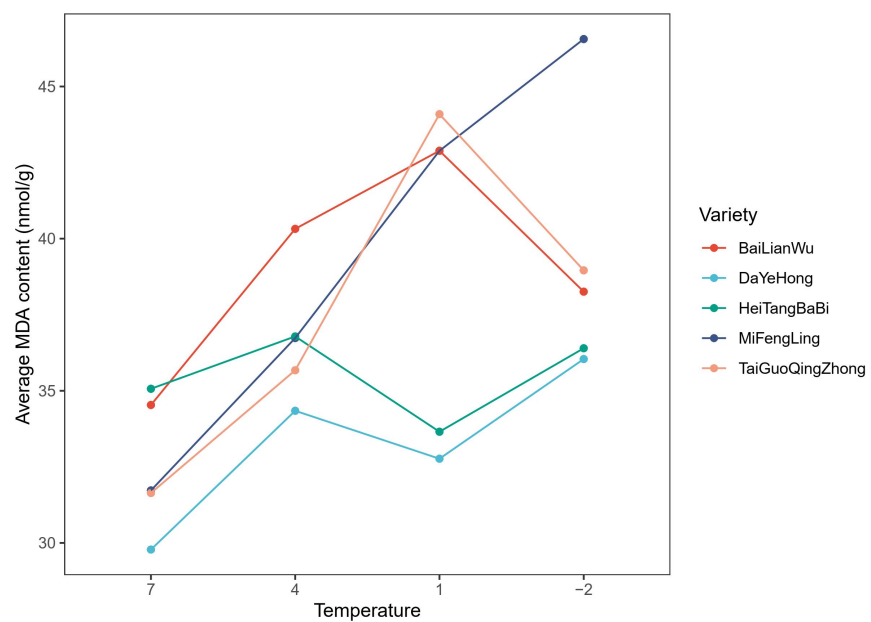

C

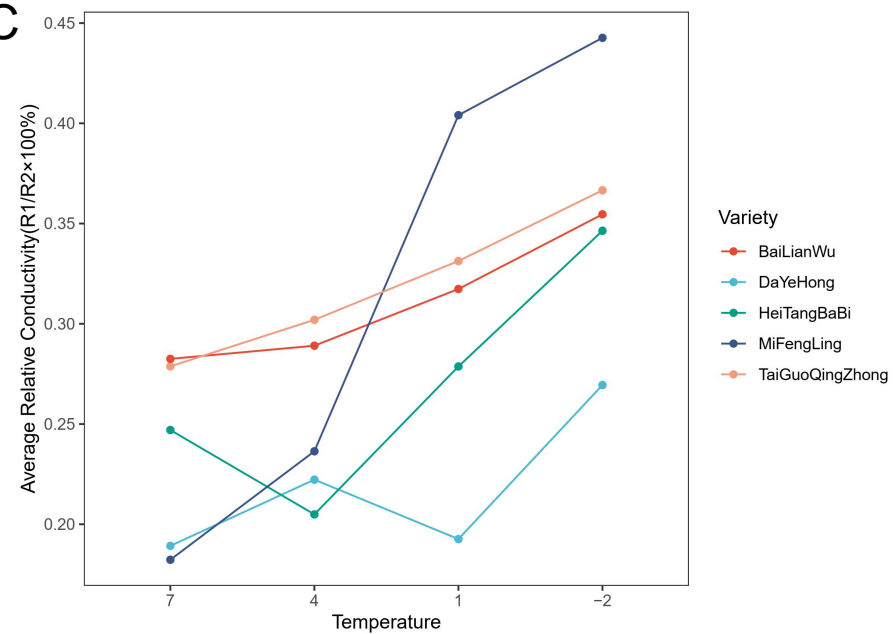

Supplement: Supplementary Figure 3 — Cold tolerance assessment of wax apple. (A) Phenotypic responses of wax apple leaves under cold stress treatment. (B) MDA content in wax apple leaves under cold stress. (C) Relative electrolyte conductivity of wax apple leaves under cold stress. [file DataSheet3.pdf]

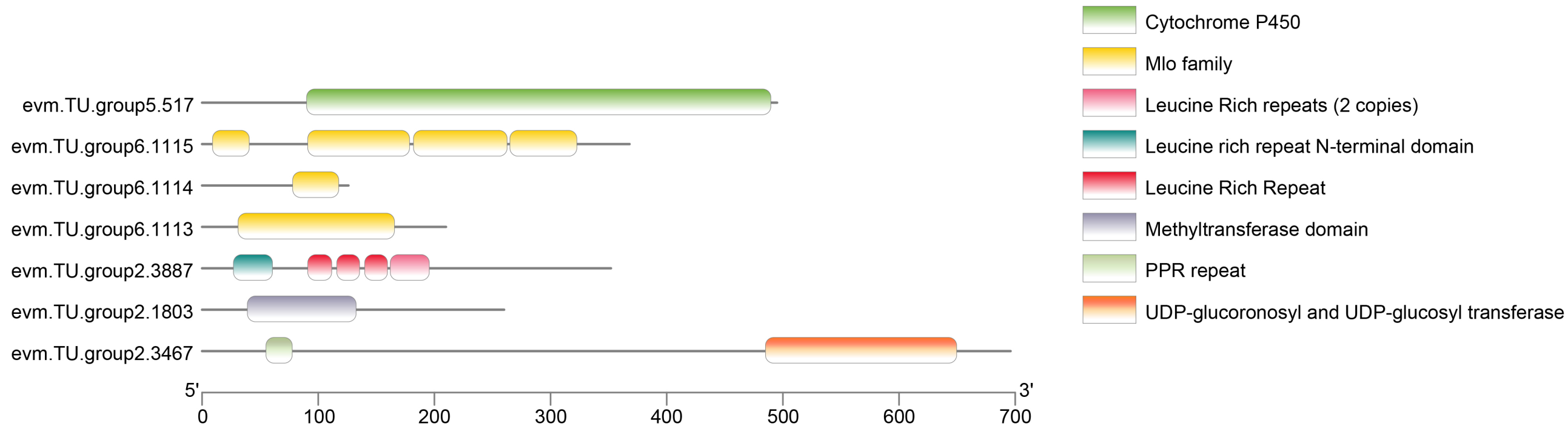

Supplement: Supplementary Figure 4 — Gene structure of fruit size and cold tolerance candidates. [file DataSheet4.pdf]

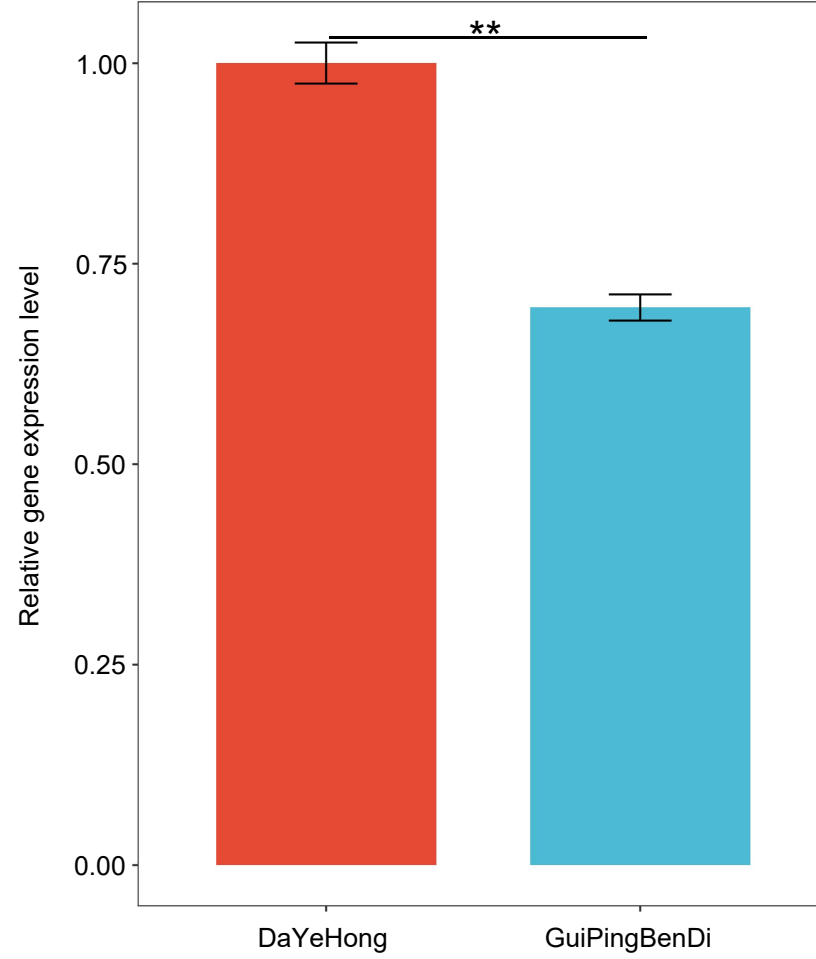

Supplement: Supplementary Figure 5 — The expression level of evm.TU.group6.1115 in the species with different fruit size. ** represents that there is a significant change in the two samples. [file DataSheet5.pdf]
